# Activity and stability origin of core–shell catalysts: unignorable atomic diffusion behavior†

**DOI:** 10.1039/d4sc08019j

**Published:** 2025-01-13

**Authors:** Yuanyuan Xue, Letian Chen, Lijuan Zhang, Gengfeng Zheng, Xu Zhang, Zhen Zhou

**Affiliations:** a Laboratory of Advanced Materials, Department of Chemistry and Shanghai Key Laboratory of Molecular Catalysis and Innovative Materials, Fudan University Shanghai 200438 China gfzheng@fudan.edu.cn; b Interdisciplinary Research Center for Sustainable Energy Science and Engineering (IRC4SE^2^), School of Chemical Engineering, Zhengzhou University Henan 450001 China zzuzhangxu@zzu.edu.cn; c School of Materials Science and Engineering, Institute of New Energy Material Chemistry, Renewable Energy Conversion and Storage Center (ReCast), Key Laboratory of Advanced Energy Materials Chemistry (Ministry of Education), Nankai University Tianjin 300350 China zhouzhen@nankai.edu.cn

## Abstract

The exceptional oxygen reduction reaction (ORR) and oxygen evolution reaction (OER) performances of core–shell catalysts are well documented, yet their activity and durability origins have been interpreted only based on the static structures. Herein we employ a NiFe alloy coated with a nitrogen-doped graphene-based carbon shell (NiFe@NC) as a model system to elucidate the active structure and stability mechanism for the ORR and OER by combining constant potential computations, *ab initio* molecular dynamic simulations, and experiments. The results reveal that the synergistic effects between the alloy core and carbon shell facilitate the formation of Fe–N–C active sites and replenish metal sites when central metal atoms detach. The metal core and catalytic environment function as an “ammunition depot” and “automatic loader,” respectively, ensuring long-term stability. Notably, atomic diffusion behaviors are identified as critical for the formation and regeneration of active sites during the ORR/OER. This work provides new insights into the activity and stability of core–shell catalysts and emphasizes the importance of reconstruction and dynamic structural evolution in electrocatalysts.

## Abstract 2

The dynamic evolution of a core–shell catalyst (M@C) is revealed *via* computations and experiments, showing that metal atoms diffuse to form M–N–C sites, which are highly active and capable of regenerating for oxygen reduction and evolution reactions.

## Graphical abstract

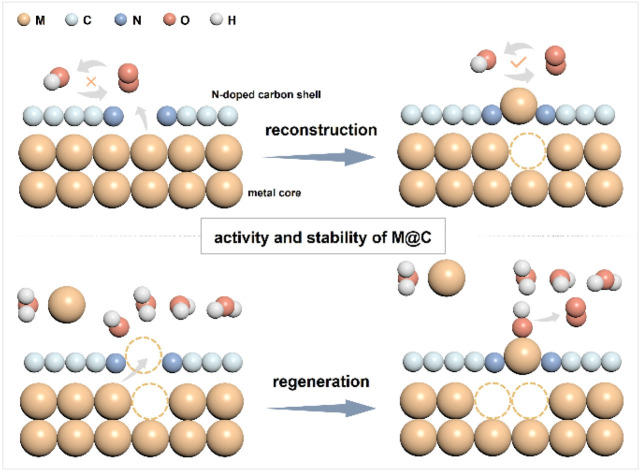

## Introduction

Core–shell catalysts, sometimes referred to as chainmail catalysts,^1–3^ excel in energy-related electrochemical processes,^4^ with transition metals or alloys coated with graphene-based carbon shells (M@C) exhibiting exceptional electrocatalytic activity and enduring stability for the oxygen reduction reaction (ORR) and oxygen evolution reaction (OER) under alkaline environments.^5–9^ Most studies have suggested that the activation of carbon shells occurs through the electron transfer from transition metal atoms to the outer carbon shells, where the reactions predominantly occur.^10,11^ Meanwhile, the carbon shells also act as a protective layer for the inner transition metals or alloys.^10^ Some studies indicate that reactants can penetrate the porous carbon shell so that the reactions directly proceed on metal or alloy surfaces.^12^ However, the structure reconstructions and dynamic evolutions of catalysts during preparation and working conditions are not considered in both views. In early 2019, Fan *et al.* reported the transformation of residual Ni particles in carbon nanotubes into thermally stable isolated single Ni atoms, which might contain a NiN_3_ moiety.^13^ Additionally, the evolution from the preformed small Fe nanoparticles to single Fe atom sites (FeN_4_) was disclosed by monitoring the pyrolytic process for preparing the Fe–N–C material with *in situ* technologies.^14^ During the preparation of M@C, carbon shells are commonly defective and doped with heteroatoms (N, P, S, O, *etc.*).^15^ Thus, in the case of M@C, the metal atoms possibly diffuse into the X-doped carbon layers and form the M–X–C sites, which are known as highly active for electrocatalytic reactions.^16–20^

Although significant progress has been made in experimental characterization at the atomic level,^21–23^ it is particularly challenging to catch the dynamic structure evolution of catalysts and identify the composites with multi-scale species, such as the composite of metal nanoparticles and single metal atoms. On the other hand, advanced computational methods can provide strong toolkits for studying catalyst reconstructions and dynamic evolution behaviors under reaction environments.^24–27^ Herein, as an example, the reconstruction of a NiFe alloy coated with a nitrogen-doped graphene-based carbon shell (NiFe@NC) is explicitly demonstrated by the combination of computations and experiments. Furthermore, the activity origin for the ORR/OER is thoroughly explored by constant potential calculations combined with implicit solvent models and the regeneration of active sites within NiFe@NC under the reaction environment is discovered using *ab initio* molecular dynamic (AIMD) simulations combined with explicit solvent models. These findings allow to deepen the understanding of the activity and stability origins of core–shell catalysts under electrochemical conditions.

## Results and discussion

### Diffusion of transition metal atoms

**Fig. 1:**
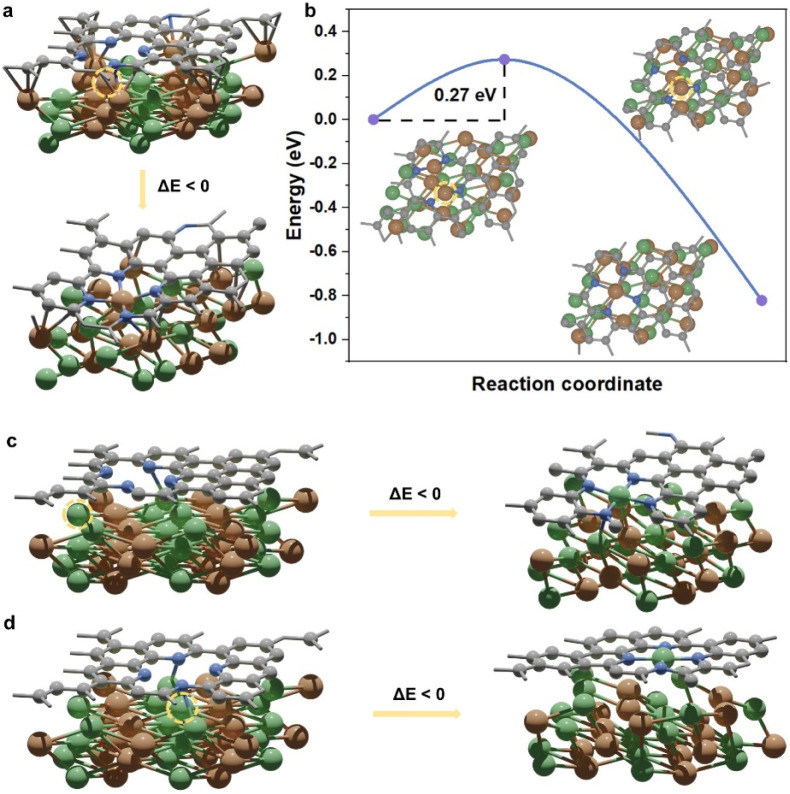
(a) The initial state and optimized final state of NiFe_Fe_up@NC-A. (b) The energy profile for the structure reconstruction of NiFe_Fe_up@NC-B, and corresponding structures of the initial state, the transition state, and the final state. (c) The initial state and optimized final state of NiFe_Ni_up@NC-A. (d) The initial state and optimized final state of NiFe_Ni_up@NC-B. Color representations: orange, Fe; green, Ni; blue, N; gray, C. The color representations below are the same as there. Dashed circles highlight the diffusing metal atoms.

For a comprehensive analysis, four NiFe@NC structures have been considered (Fig. S1†), and are denoted as NiFe_Fe_up@NC-A, NiFe_Fe_up@NC-B, NiFe_Ni_up@NC-A, and NiFe_Ni_up@NC-B. They can be classified into two types: (I) iron terminated alloy surface, and (II) nickel terminated alloy surface. Each type exhibits two distinct locations for four pyridinic nitrogen atoms. Specifically, for type (I), the Fe–N–C site spontaneously forms at location A (Fig. 1a). Despite the presence of a barrier to the formation of the Fe atom at location B (Fig. 1b), the barrier is sufficiently small (0.27 eV) to be overcome under real preparation and working conditions. On the other hand, for type (II), the Ni–N–C site spontaneously forms both at location A and location B (Fig. 1c and d). Consequently, during the preparation of alloy@NC, there is a high possibility of metal atoms diffusing from the inner alloy to the outer nitrogen-doped carbon shell, and the M–N–C sites form at the N-doped carbon shell.

**Fig. 2:**
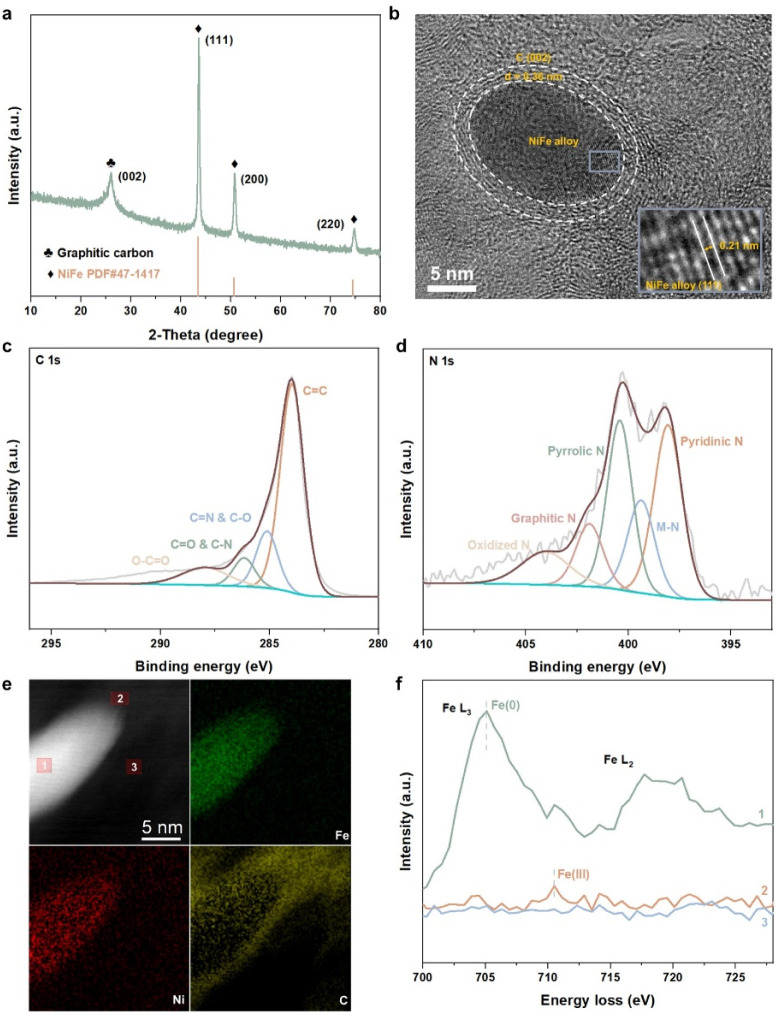
(a) X-ray diffraction (XRD) pattern of NiFe@NC-sys. (b) High-resolution transmission electron microscopy (HRTEM) image of NiFe@NC-sys. (c) High-resolution C 1s XPS of NiFe@NC-sys. (d) High-resolution N 1s XPS of NiFe@NC-sys. (e) AC-STEM image and corresponding EELS mapping of NiFe@NC-sys. (f) EELS profiles from three positions marked in the AC-STEM image of (e).

For further confirmation of the above results, we synthesized a sample comprising NiFe alloy nanoparticles coated by nitrogen-doped carbon shells (NiFe@NC-sys) by high-temperature pyrolysis (details in ESI†). The strongest X-ray diffraction peak of NiFe@NC-sys is attributed to the (111) crystal plane of the NiFe alloy with an atomic ratio of 1 : 1 (Fig. 2a), which is consistent with the computational models employed in this work. In NiFe@NC-sys, the NiFe alloy nanoparticles are supported on the graphitic carbon network (Fig. S2†). Furthermore, there are very thin (2–3 nm) graphitic carbon shells surrounding the NiFe alloy nanoparticles (Fig. 2b), confirming the successful preparation of the core–shell structure. The atomic ratio between Fe and Ni within NiFe@NC-sys is further determined to be 1 : 1 by X-ray photon electron spectroscopy (XPS) (Table S1†). The peaks at 286.2 eV and 285.2 eV from high-resolution C 1s XPS of NiFe@NC-sys (Fig. 2c) indicate that nitrogen atoms have been doped into the graphitic carbon skeleton. Moreover, the high-resolution N 1s XPS (Fig. 2d) obviously shows the peak (399.4 eV) corresponding to the M–N coordination structure, suggesting the existence of Fe–N–C and Ni–N–C. There is the most pyridinic nitrogen (398.1 eV) in different nitrogen species included in NiFe@NC-sys (Fig. S3†) and we also employed the pyridinic nitrogen-doped carbon structure in the computations.

To further reveal the structure reconstruction during the preparation of NiFe@NC-sys, spherical-aberration corrected scanning transmission electron microscopy (AC-STEM) and electron energy loss spectroscopy (EELS) were used. The distributions of Fe and Ni within the nanoparticle of NiFe@NC-sys are uniform as displayed in Fig. 2e. More importantly, the EELS profiles of the iron element extracted from the alloy (position 1), the carbon shell (position 2), and the carbon substrate (position 3) show the difference (Fig. 2f). Compared with the peak corresponding to Fe(0) of the alloy, the EELS profile at the carbon shell shows a peak at higher energy, indicating the formation of Fe–N–C at the carbon shell. The difference in peak location confirms the computational results, which proposes the diffusion of Fe atoms from the inner NiFe alloy to the outer carbon shell and the reconstruction of NiFe@NC during preparation. On the other hand, there is no obvious signal attributed to the Fe element at the carbon substrate. Using *operando* electrochemical scanning tunneling microscopy, Kosmala *et al.*^28^ found that in thin iron films covered with monolayer graphene, single iron atoms could be captured by graphene vacancies under reaction conditions, and the formed isolated Fe sites are active for the hydrogen evolution reaction. In our work, the doped nitrogen species rather than the vacancies within the carbon structure serve to anchor Fe atoms diffusing from the metal/alloy.

### Origin of the reaction activity

Then the ORR and OER performances on the reconstructed NiFe@MNC were explored. Here, M refers to Fe or Ni, representing that one metal atom (Fe or Ni) of the NiFe alloy migrates to the outer nitrogen-doped carbon shell and forms the M–N–C site. The ORR and OER paths were calculated using the constant potential method with an implicit solvent model (computational details in ESI†). For comparison, the ORR and OER activities of unreconstructed NiFe@NC (Fig. S4†) were also calculated, in which the NiFe alloy is coated by a nitrogen-doped carbon shell and no M–N–C site exists on the carbon shell, which is generally considered to be the active structure for M@C catalysts.^10,11^ Previous reports claimed that the electron transfer from the metal core to the outer nitrogen-doped carbon shell is the key factor for the significant ORR and OER performances of M@C catalysts, with carbon atoms adjacent to nitrogen as the adsorption sites of oxygen-containing intermediates and reaction sites.^10,11^

**Fig. 3:**
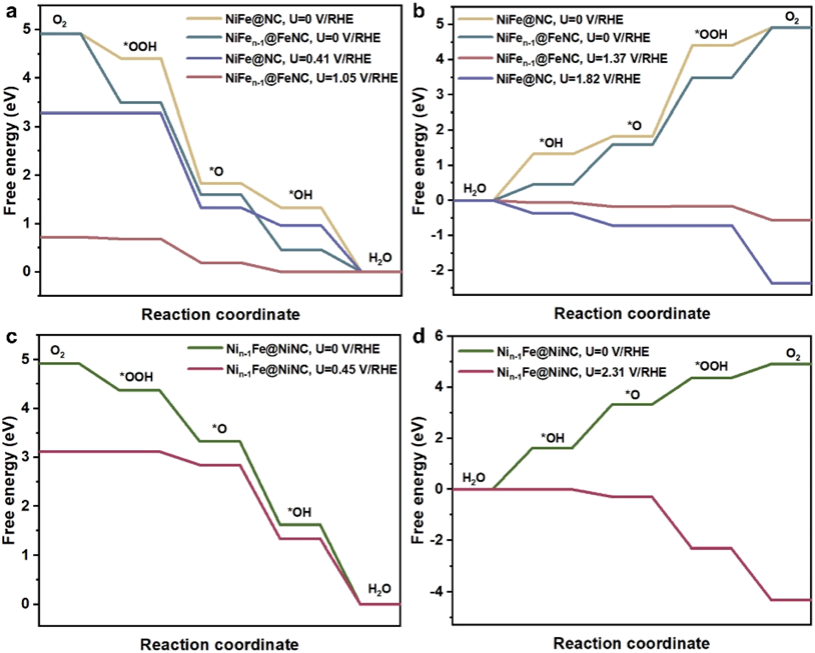
(a) The free energy profiles for the ORR on NiFe_*n*−1_@FeNC and unreconstructed NiFe@NC at pH = 13. (b) The free energy profiles for the OER on NiFe_*n*−1_@FeNC and unreconstructed NiFe@NC at pH = 13. (c) The free energy profiles for the ORR on Ni_*n*−1_Fe@NiNC at pH = 13. (d) The free energy profiles for the OER on Ni_*n*−1_Fe@NiNC at pH = 13.

Interestingly, the onset potentials of NiFe_*n*−1_@FeNC for the ORR and OER are superior to those of unreconstructed NiFe@NC (Fig. 3a and b, computational details in Fig. S5a, b and Tables S2–S4†), and are well consistent with reported onset potentials of similar core–shell catalysts comprising a NiFe alloy coated with a nitrogen-doped carbon shell under the same pH conditions (Table S5†), suggesting that Fe–N–C sites formed after the diffusion of Fe atoms are likely to serve as active sites for the ORR and OER, rather than only the NiFe alloy indirectly influencing the performances of the ORR and OER through the carbon shell. Moreover, compared with the pyridine N-coordinated Fe–N–C alone,^16^ the presence of alloy particles greatly improves the ORR reactivity of the pyridine N-coordinated Fe–N–C site (Table S6†). Electrons of the outermost Fe and Ni atoms within the NiFe alloy are transferred to the Fe–N–C site (Fig. S6a and b†), and the charge density of the central Fe atom in the Fe–N–C site increases by 0.34 e Å^−1^ (Fig. S6c and d†). The synergy between the Fe–N–C site and the inner NiFe alloy contributes to the enhanced ORR and OER performances. The potential-dependent and pH-dependent adsorption energies of intermediates (*OH, *O, and *OOH) on NiFe_*n*−1_@FeNC were analyzed to further disclose the activity mechanism. At pH = 13, the rate-determining step (RDS) of the ORR on NiFe_*n*−1_@FeNC is the removal of *OH (Fig. 3a). As shown in Fig. S7a,† the binding strength of *OH increases with decreasing the potential. Thus, the formation of *OH is easier when the potential is more negative than the onset potential (1.05 V *vs.* RHE, RHE refers to the reversible hydrogen electrode). On the other hand, the binding strength decreases for *OH, *O, and *OOH with increasing the potential (Fig. S7b–d†), and the binding strength of *OOH is obviously weaker than that of *O (Fig. S7b†). Thus, at pH = 13, the RDS of the OER on NiFe_*n*−1_@FeNC is the transformation of *O to *OOH (Fig. 3b).

The ORR onset potential of Ni_*n*−1_Fe@NiNC is close to that of unreconstructed NiFe@NC, while the OER onset of Ni_*n*−1_Fe@NiNC is higher than that of unreconstructed NiFe@NC (Fig. 3c and d, computational details in Fig. S5c, Tables S2 and S7†). Ni single-atom catalysts are poorly active for the ORR, and most related studies have revealed that Ni–Fe dual-atom catalysts are active for the OER instead of Ni single-atom catalysts.^29–32^ The OER and ORR onset potentials of Ni_*n*−1_Fe@NiNC are poorer than those of NiFe_*n*−1_@FeNC, which indicates that Fe plays a more critical role than Ni in Fe–Ni bimetallic-based catalysts.^33^

### Stability mechanism

**Fig. 4:**
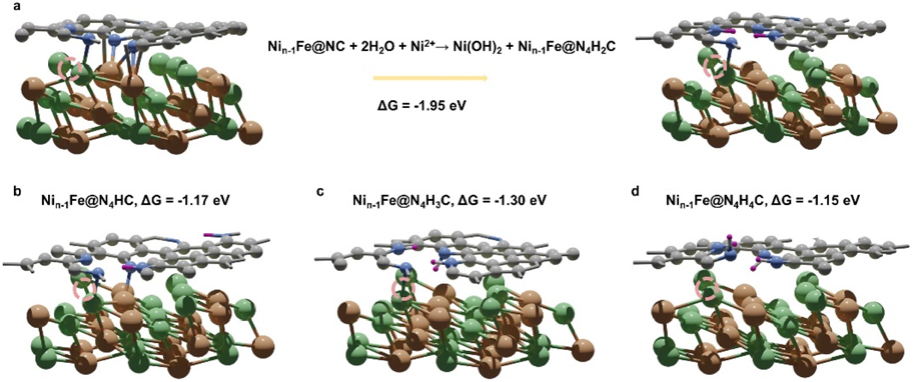
(a) The transformation from Ni_*n*−1_Fe@NC to the preferable protonation structure Ni_*n*−1_Fe@N_4_H_2_C and the corresponding protonation free energy change of this process. (b–d) Other possible protonation structures of Ni_*n*−1_Fe@NC and corresponding protonation free energy changes: (b) Ni_*n*−1_Fe@N_4_HC, (c) Ni_*n*−1_Fe@N_4_H_3_C, and (d) Ni_*n*−1_Fe@N_4_H_4_C. The dark purple spheres represent hydrogen atoms. Dashed circles highlight the original places of detached Ni atoms.

M@C catalysts not only have the synergistic catalytic effect of their core and shell, but also the presence of carbon shells can effectively improve the stability of catalysts.^10^ Previous work has indicated that the shell can avoid direct contact between vital metals/alloys and harsh reaction conditions.^10^ Here the stability of NiFe@MNC is also considered. Both computational and experimental studies have revealed that the central metal atoms at the M–N–C sites are possible to dissolve during the ORR/OER in alkaline environments under the working potentials.^34,35^ This research reveals that when the central metal atom of the M–N–C site detaches from the carbon shell of the NiFe@MNC catalyst, its activity and stability depend on the type of the central metal. For Ni_*n*−1_Fe@NiNC, a more stable Ni_*n*−1_Fe@N_4_H_2_C structure forms subsequent to the loss of the center Ni atom (Ni_*n*−1_Fe@NiNC + 2H_2_O → Ni(OH)_2_ + Ni_*n*−1_Fe@N_4_H_2_C) (Fig. 4a), indicating that once the Ni atom of the Ni– N–C site leaves, the active Ni–N–C site is damaged. Other possible protonation structures from the degradation of Ni_*n*−1_Fe@NiNC are also shown in Fig. 4b–d, and the protonation free energy changes for those structures are more negative than −1 eV, suggesting the instability of Ni_*n*−1_Fe@NiNC.

**Fig. 5:**
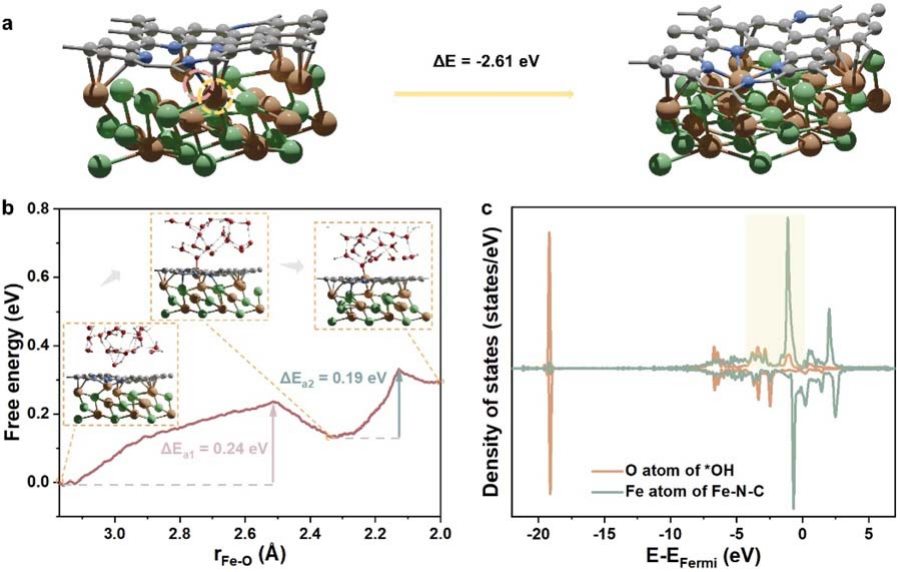
(a) The structure transformation of NiFe_*n*−1_@NC. The pink circle highlights the original place of the detached Fe atom. The yellow circle highlights the diffusing Fe atom. (b) The free energy profile obtained from AIMD. The red spheres, white spheres, and dashed lines in the insets represent O atoms, H atoms, and hydrogen bonds, respectively. (c) The projected density of states (PDOS) of the final structure of stage 1, the structure is inserted in (b).

Contrastively, when the center Fe atom of the Fe–N–C departs from NiFe_*n*−1_@FeNC, the Fe atom from the inner NiFe alloy migrates spontaneously to the nitrogen-doped carbon surface, leading to the reformation of the Fe–N–C site (Fig. 5a). In other words, the Fe–N–C site of NiFe_*n*−1_@FeNC is regenerative. Thus, the highly active NiFe_*n*−1_@FeNC also exhibits impressive stability owing to the continuous regeneration of the Fe–N–C sites. The AIMD simulations with an explicit solvent model were performed to further understand the dynamic-regeneration process of the Fe–N–C sites within NiFe_*n*−1_@FeNC. The initial state corresponds to the transformed structure in Fig. 5a and 48 water molecules and 1 hydroxide are introduced. The hydroxide is involved at the interface between the slab and water molecules to realistically simulate the alkaline reaction environment. The “slow growth” method is adopted, and the distance between the outermost Fe atom of the NiFe_*n*−1_ alloy and the O atom of hydroxide is constrained (the detailed setting of the collective variable in ESI†). During the dynamic process, two minor barriers are identified (Fig. 5b), where the first barrier (0.24 eV) originates from the continuous diffusion of the outermost Fe atom toward the outer carbon shell (stage 1). The hydroxide is adsorbed on this Fe atom at the end of stage 1. For the final structure of stage 1, the orbitals of the O atom from *OH showcase intense interactions with those of the Fe atom from the Fe–N–C site (Fig. 5c and S8†). The second barrier (0.19 eV) arises from the continuous outward migration of the Fe atom from the Fe–N–C site with the assistance of *OH. Hydroxide adsorption plays an auxiliary role in the regeneration of active sites of NiFe_*n*−1_@FeNC.

Since the simulated cell size is limited, the free energy changes and barriers obtained directly from the AIMD simulation are determined at different potentials. Nevertheless, the ORR and OER are conducted under constant potentials. In this case, the free energy changes and barriers of the dynamic process under 0 V_RHE_, 0.8 V_RHE_, and 1.5 V_RHE_ at pH = 14 were calculated ulteriorly, based on the constant potential correction^36^ and charge-extrapolation method.^37^ Specifically, a potential of 0.8 V_RHE_ corresponds to the working potential for the ORR, while 1.5 V_RHE_ represents the working potential for the OER. Compared with the barriers and free energy changes under 0 V_RHE_, especially the free energy change and barrier of stage 1 are close to 0 eV at 0.8 V_RHE_, and even become negative at 1.5 V_RHE_ (Table S10, computational details in Tables S8 and S9†), demonstrating the ease of Fe atom diffusion. The barriers of stage 1 and stage 2 are smaller than 0.3 eV under working potentials and can be easily overcome. Therefore, the synergistic effects between the metal core and carbon shell not only enable the *in situ* generation of M–N–C active sites, but also replenish the metal active sites to enhance the overall stability. Importantly, the hydroxide adsorption, as a critical intermediate step in the ORR/OER, also facilitates the regeneration of metal active sites. During the catalytic ORR/OER processes, the metal core acts as the “ammunition depot”, alkaline environments and working potentials act as the “automatic loader”, which collectively ensure the exceptional long-term stability of the core–shell catalysts under working conditions.

## Conclusions

In conclusion, the detailed catalytic and stability mechanisms of NiFe@NC during the ORR/OER have been disclosed by using constant potential calculations and AIMD simulations combined with experimental confirmation. The diffusion of metal atoms from the inner NiFe alloy and the formation of the M–N–C site at the outer nitrogen-doped carbon shell interpret why NiFe@NC exhibits exceptional ORR and OER activities. Furthermore, the regeneration capability of the M–N–C site is found to be vital for the long-term stability of M@C during the electrocatalytic processes, and thus NiFe@NC functions as an “automatic rifle” during the ORR/OER. Our findings can provide fresh perspectives toward understanding the activity and stability origin of core–shell catalysts, and underline the significance of catalyst reconstruction and dynamic evolution under realistic reaction environments.

## Data availability

The original data supporting this article are available in the main context and ESI.†

## Author contributions

Y. Xue and X. Zhang designed the research. L. Zhang, G. Zheng, X. Zhang, and Z. Zhou supervised the research. Y. Xue performed the research and analyzed data. L. Chen contributed data processing scripts. Y. Xue, G. Zheng, X. Zhang, and Z. Zhou wrote the paper.

## Conflicts of interest

There are no conflicts to declare.

## Supplementary Material

SC-016-D4SC08019J-s001
