# Supplementary material for: Activity and stability origin of core–shell catalysts: unignorable atomic diffusion behavior
Source: Chem Sci. 2025 Jan 13;16(7):3323–8. doi: 10.1039/d4sc08019j (PMC11748047; doi:10.1039/d4sc08019j)
Supplement: SC-016-D4SC08019J-s001 [file SC-016-D4SC08019J-s001.pdf]

Supplementary Information (SI) for Chemical Science.
This journal is © The Royal Society of Chemistry 2025

Electronic supplementary information (ESI)

Activity and stability origin of core-shell catalysts: Unignorable atomic diffusion behavior

Yuanyuan Xue,[a] Letian Chen,[c] Lijuan Zhang,[a] Gengfeng Zheng,*[a] Xu Zhang*[b] and Zhen Zhou*[b,c]

[a] *Laboratory of Advanced Materials, Department of Chemistry and Shanghai Key Laboratory of Molecular Catalysis and Innovative Materials, Fudan University, Shanghai 200438, China*

[b] *Interdisciplinary Research Center for Sustainable Energy Science and Engineering (IRC4SE^2), School of Chemical Engineering, Zhengzhou University, Henan 450001, China*

[c] *School of Materials Science and Engineering, Institute of New Energy Material Chemistry, Renewable Energy Conversion and Storage Center (ReCast), Key Laboratory of Advanced Energy Materials Chemistry (Ministry of Education), Nankai University, Tianjin 300350, China*

*Corresponding author. Email: gfzheng@fudan.edu.cn (G.F.Z.); zzuzhangxu@zzu.edu.cn (X.Z.); zhouzhen@nankai.edu.cn (Z.Z.).

Table of Contents

Computational Procedures **1**
Modeling 1
Constant potential calculations 2
AIMD simulations 3
Experimental Procedures **4**
Preparation 4
Characterization 5
Supplemental Figures and Tables **6**
Figure S1 6
Figure S2 7
Figure S3 8
Figure S4 9
Figure S5 10
Figure S6 11
Figure S7 12
Figure S8 13
Table S1 14
Table S2 15
Table S3 16
Table S4 17
Table S5 18
Table S6 19
Table S7 20
Table S8 21
Table S9 22
Table S10 23
References **24**

Computational Procedures

Modeling

DFT computations with spin-polarization are carried out using the Vienna Ab initio Simulation Package (VASP).[1] The Perdew-Burke-Enrzerhof (PBE) parametrization of the generalized gradient approximation (GGA) is used to describe the electronic exchange-correlation energies.[2] The dispersion correction (D3-Grimme) is employed to better describe the weak interactions.[3] Three layers of NiFe alloy (111) plane (mp-2213, the ratio between Fe and Ni is 1:1) and one graphene layer doped with four pyridinic N are combined to simulate NiFe@NC. The graphene layer contains 28 of C atoms and 4 of pyridinic N atoms. A vacuum space of more than 15 Å is employed along the z-axis to avoid the interaction between two periodic units. When relaxing, the bottom layer is fixed to simulate the bulk NiFe alloy. Brillouin zones are sampled by a Gamma-centered Monkhorst-Pack k-point mesh with 2 × 3 × 1 for NiFe@NC. During the structure relaxation, the setup of convergence criterion for residual force and energy are 0.03 eV/Å and 10^{-5} eV, respectively. An energy cutoff of 400 eV for the plane-wave basis set is used.

To calculate the barrier for the formation of NiFe-(n-1)@FeNC from NiFe@NC, the climbing image nudged elastic band method (CINEB) method[4] is used to search the transition state and further the DIMER method[5] is adopted to obtain a more accurate energy of the transition state. Both methods adopt 10^{-7} eV and 0.03 eV/Å standards for energy and force convergence, respectively. Other parameter settings are the same as the before mentioned.

Constant potential calculations

When conducting constant potential calculations, the implicit solvent environment is presented by the VASPsol code.[6,7] The relative permittivity is set to 80 to model the aqueous electrolyte. The effective surface tension parameter uses 0 to neglect the cavitation energy contribution. The linearized Poisson−Boltzmann model with a Debye length of 3.0 Å mimics the compensating charge. To consider the effect of electrode potentials during ORR/OER processes, the excess charge is added to the unit cell, the value (Δn) is varied from −2.0 e to +2.0 e in steps of 0.5 e/0.25 e for $NiFe_{n-1}$@FeNC, Δn is varied from −6.0 e to −2.0 e in steps of 0.5 e for $Ni_{n-1}Fe$@NiNC, and Δn is varied from −6.0 e to 0.0 e in steps of 0.5 e for unreconstructed NiFe@NC. Then the energy (E) of the system is related to the potential, and the relationship between E and Δn follows Equation 1. The electrode potential (U_q) is referenced to the standard hydrogen electrode (SHE) according to Equation 2. Finally, $E-U_q$ follows a quadratic function relationship (Equation 3).[8]

$$E = E_{DFT} - \Delta n(V_{sol} + \Phi_q/e) \quad (1)$$

$$U_q(V_{SHE}) = -4.6\ V - \Phi_q/e \quad (2)$$

$$E = -1/2C(U_q - U_0)^2 + E_0 \quad (3)$$

In Equation 1, E_{DFT} is the energy calculated by VASP; another term is the energy correction of the charged system, where V_{sol} refers to the electrostatic potential of the bulk solution, and $-\Phi_q$ represents the work function. In Equation 2, 4.6 V is the work function of the H_2/H^+ couple at standard conditions. In Equation 3, C, U_0, and E_0 are

the capacitance, the potential of zero charge (PZC), and the corresponding energy of the system at PZC, respectively.

The calculated ORR steps are listed as follows. The sequence of OER steps is reversed from those of ORR. The free energy changes (ΔG) of elementary steps are calculated as $\Delta G = \Delta E + \Delta E_{ZPE} - T\Delta S$, where ΔE, ΔE_{ZPE}, T, and ΔS are the reaction energy, zero-point energies correction, temperature, and entropy change, respectively. The free energy correction of adsorbed intermediates is calculated based on the VASPKIT tool at 298.15 K.[9] ΔE is calculated by the double reference method, which is potential dependent. The chemical potential of the solvated proton and electron pair is equal to $1/2G_{H2} + eU - 0.059pH$.

$$* + O2 + H2O + e^- \rightarrow *OOH + OH^- \quad (4)$$

$$*OOH + e^- \rightarrow *O + OH^- \quad (5)$$

$$*O + H2O + e^- \rightarrow *OH + OH^- \quad (6)$$

$$*OH + e^- \rightarrow * + OH^- \quad (7)$$

AIMD simulations

To clarify the dynamic structure evolution of NiFe@NC during realistic reaction environments, we performed AIMD simulations with explicit solvent environments using VASP.[1] 48 water molecules (1 g cm^{-3} of density) are introduced into the simulated system. One hydroxide is involved at the interface between the slab and the water molecules for simulating the reaction environments since M@C catalysts generally work under alkaline environments. The height of the vacuum layer is 10 Å to avoid the periodic image interaction.

The PBE parametrization of the GGA is used to describe the electronic exchange-correlation energies.[2] And the dispersion correction (D3-Grimme) is employed to better describe the weak interactions.[3] The cutoff energy is 400 eV for AIMD simulations. The "slow growth" method is adopted to obtain the free energy profile of dynamic behaviors of $NiFe_{n-1}$@NC. The distance between the outermost Fe atom of $NiFe_{n-1}$ alloy and the O atom of hydroxide is constrained as a collective variable (denoted as r_{Fe-O}), r_{Fe-O} is changed slowly from 3.2 Å to 2.0 Å with a change of -0.0004 Å every step. The step is set to be 1 fs. The simulation is conducted under an NVT ensemble at a constant 300 K using a Nose-Hoover thermostat.[10,11] Only the gamma point is sampled at the first Brillouin zone when simulating since the simulated system is relatively huge.

The free energy barriers and free energy changes directly obtained from the AIMD simulation are under changeable potentials, due to the limited size of the simulated system. Thus, the constant potential correction method[12] and charge-extrapolation method[13] are used to calculate the free energy barriers and free energy changes of the dynamic process under the working potential. Bader charges of the slabs are calculated to analyze the internal charge transfers of the periodic cells.

Experimental Procedures

Preparation

Iron acetate ($Fe(CH_3COO)_2$), nickel acetate titrate ($Ni(CH_3COO)_2 \cdot 4H_2O$), melamine ($C_3H_6N_6$), and glucose ($C_6H_{12}O_6$) are purchased and directly used in the preparation. Firstly, 0.05 g of iron acetate, 0.07g of nickel acetate titrate, 6 g of melamine, and 0.5 g of glucose are mixed in 100 ml of deionized water, and the suspension is stirred

magnetically for 12 h. Then the suspension is dried at 80 °C. After that, the dried powder is pyrolyzed at 900 °C for 3h under an Ar atmosphere, the rate from room temperature to 900 °C is 5 °C per minute.

Characterization

The prepared sample is characterized by an X-ray diffractometer (Bruker, D8 Discover) with Cu K_{α} radiation, a field-emission transmission electron microscope (JEOL, JEM-2100F), an X-ray photoelectron spectroscope (XPS, Thermo, Scientific K-Alpha), and a spherical-aberration corrected scanning transmission electron microscope (JEOL, JEM-ARM300F) equipped with an X-ray energy spectroscope.

Supplemental Figures and Tables

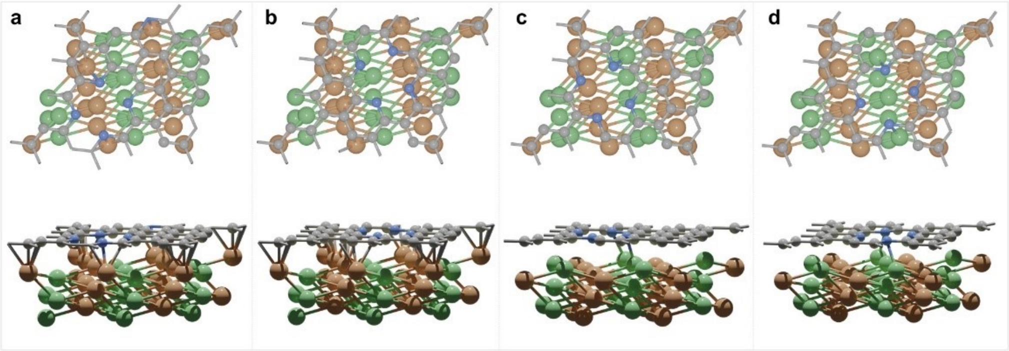

Fig. S1 (a–d) Top views and side views of (a) NiFe_Fe_up@NC-A, (b) NiFe_Fe_up@NC-B, (c) NiFe_Ni_up@NC-A, (d) NiFe_Ni_up@NC-B. Here A and B represent two kinds of locations for pyridinic nitrogen atoms. Colors: yellow, Fe; gray, Ni; brown, C; pale blue, N. The color representations below are the same as there.

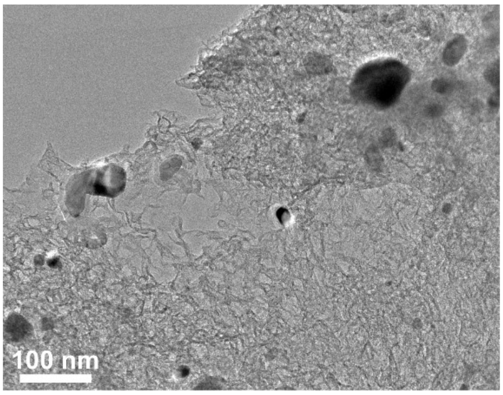

Fig. S2 Transmission electron microscopy (TEM) image of NiFe@NC-sys.

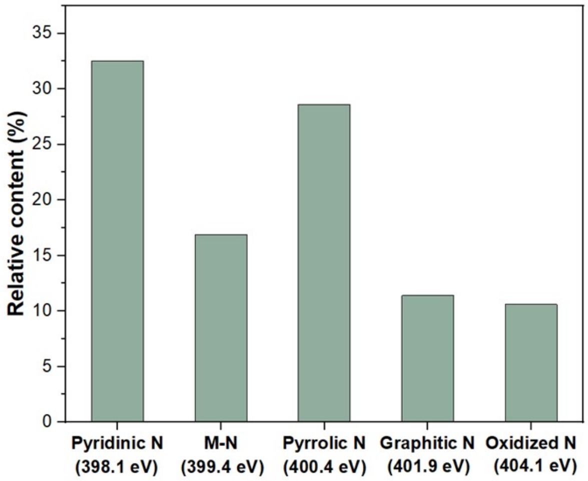

Fig. S3 The relative content of nitrogen species within NiFe@NC-sys determined by XPS. The binding energies of these nitrogen species are shown in the brackets.

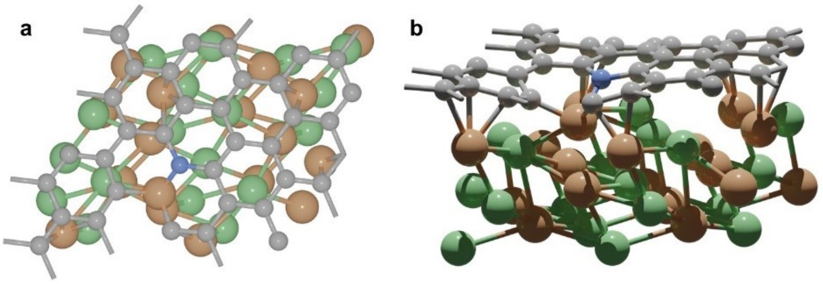

Fig. S4 Top view (a) and side view (b) of unreconstructed NiFe@NC.

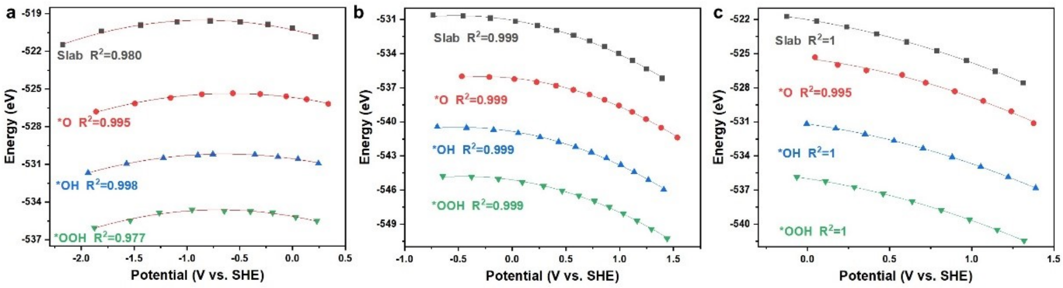

Fig. S5 (a–c) The constant potential calculation data and quadratic relationship fitting plots of (a) $NiFe_{n-1}$@FeNC, (b) unreconstructed NiFe@NC, and (c) Ni_{n-1}Fe@NiNC.

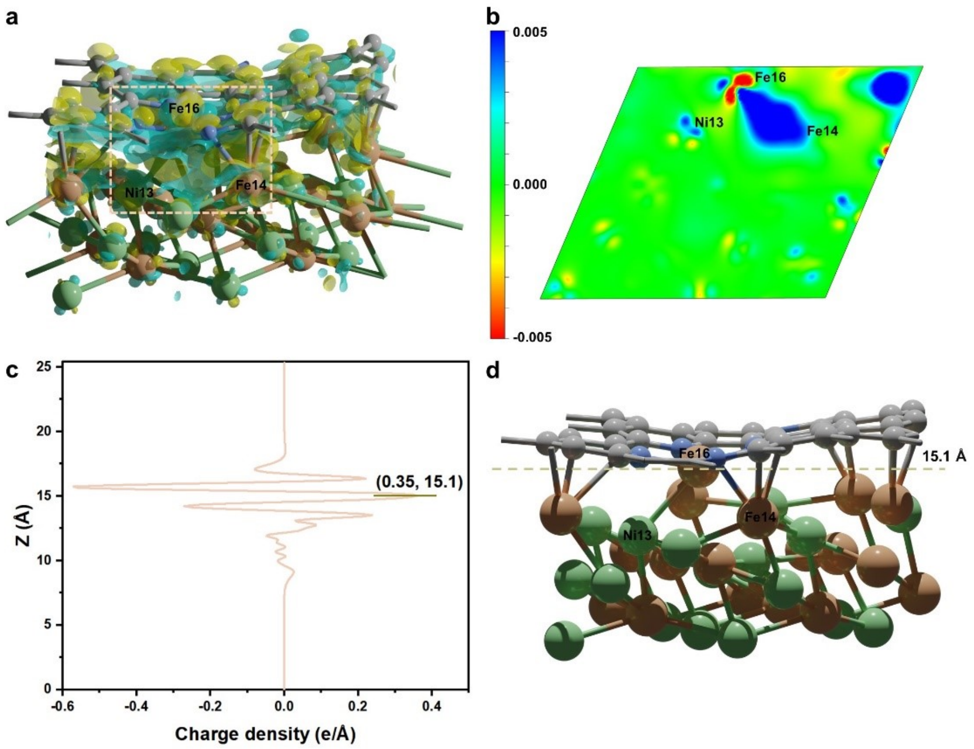

Fig. S6 (a) Charge density differential figure of $NiFe_{n-1}$@FeNC. Yellow region means the increase of charge density and cyan color means the decrease of charge density. It can be clearly seen the electron transfer from the inner $NiFe_{n-1}$ alloy to the outer N-doped carbon shell, especially to the Fe-N-C site. (b) Two-dimensional slice of the three-dimensional differential charge density of $NiFe_{n-1}$@FeNC. The electrons of the outermost Ni and Fe atoms within $NiFe_{n-1}$ alloy are transferred to the Fe-N-C site. (c) One-dimensional differential charge density (along the vacuum layer direction) of $NiFe_{n-1}$@FeNC. The charge density of the Fe atom from the Fe-N-C site increases by 0.35 e/Å. (d) The structure of $NiFe_{n-1}$@FeNC.

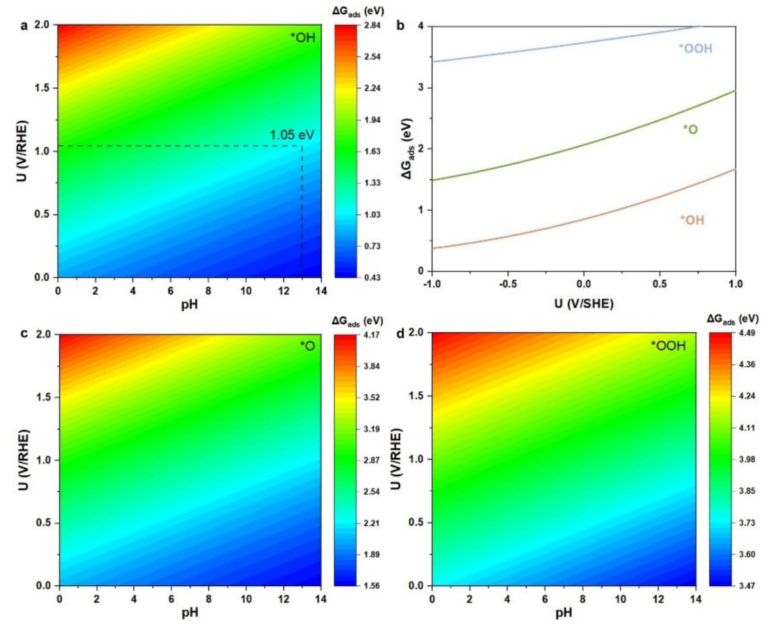

Fig. S7 (a) The pH-dependent and potential-dependent contour plot of adsorption energy of *OH on $NiFe_{n-1}$@FeNC. (b) Adsorption energies of *OOH, *O, and *OH as a function of the applied potential on $NiFe_{n-1}$@FeNC. (c) The pH-dependent and potential-dependent contour plot of adsorption energy of *O on $NiFe_{n-1}$@FeNC. (d) The pH-dependent and potential-dependent contour plot of adsorption energy of *OOH on $NiFe_{n-1}$@FeNC.

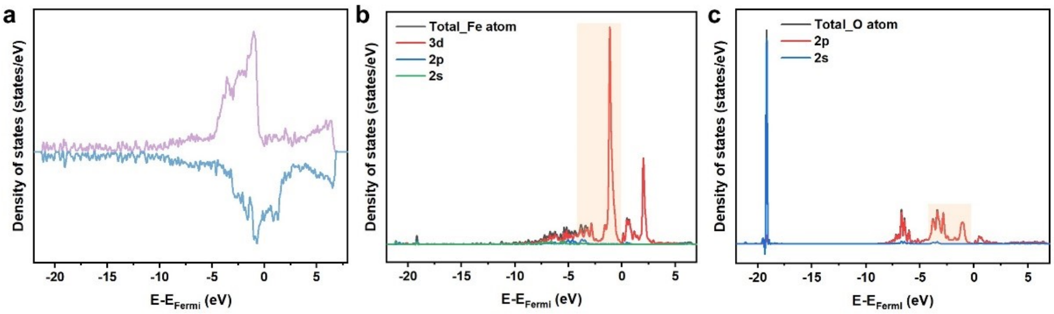

Fig. S8 (a) The spin-up and spin-down total density of states of the final structure of stage 1, the structure is inserted in Fig. 5b. (b) PDOS of the Fe atom. (c) PDOS of the O atom. The interaction between the O atom from *OH and the Fe atom from the Fe-N-C site is mainly from the contribution of Fe 3d orbitals and O 2p orbitals.

Table S1. The element content of NiFe@NC-sys.

Element	Atomic percent (%)
Ni	0.64
Fe	0.80
C	92.85
N	5.71

Table S2. The Gibbs free energies of H_2(g), O_2(g) and H_2O(l). Since O_2 is poorly described in DFT calculations, the free energy of O_2 is calculated by $G_{O2} = 2G_{H2O} - 2G_{H2} + 4.921$ eV. The DFT energies (E_{DFT}) of H_2 and H_2O are calculated in a 10 Å × 10 Å × 10 Å unit cell in vacuum.

	Pressure (atm)	Temperature (K)	E_{DFT} (eV)	ΔG (eV)	G (eV)
O_2 (g)	1	298.15			-9.910
H_2 (g)	1	298.15	-6.759	-0.047	-6.806
H_2O (l)	0.035	298.15	-14.219	-0.002	-14.221

Table S3. The fitted E (eV)-U (V vs. SHE) quadratic relationships of $NiFe_{n-1}$@FeNC.

	Fitted E-U quadratic relationships
Slab	$E = -520.282 - 1.810U - 1.074U^2$
*OH	$E = -530.541 - 1.161U - 0.902U^2$
*O	$E = -525.666 - 1.075U - 0.918U^2$
*OOH	$E = -535.129 - 1.475U - 1.054U^2$

Table S4. Fitted E (eV)-U (V vs. SHE) quadratic relationships of unreconstructed NiFe@NC.

	Fitted E-U quadratic relationships
Slab	$E = -1.476U^2 - 1.546U - 531.056$
*OH	$E = -1.493U^2 - 1.471U - 540.836$
*O	$E = -1.602U^2 - 0.909U - 536.148$
*OOH	$E = -1.518U^2 - 1.349U - 545.102$

Table S5. The ORR and OER performances of $NiFe_{n-1}$@FeNC and similar catalysts.

Catalyst	Atom ratio	ORR onset potential	OER onset potential	pH	Reference
$NiFe_{n-1}$@FeNC	Ni:Fe=1:1	1.05 V_{RHE}	1.37 V_{RHE}	13	This work
$NiFe_{n-1}$@FeNC	Ni:Fe=1:1	0.90 V_{RHE}	1.40 V_{RHE}	14	This work
NiFe@NC_X	Ni:Fe=1:2	1.03 V_{RHE}	1.46 V_{RHE}	13	ACS Catal. 2016, 6, 6335
FeNi@NC	Ni:Fe=1:1	0.97 V_{RHE}	-	13	Chin. J. Catal. 2024, 58, 206
NiFe@NBCNT	Ni:Fe=1:1	1.03 V_{RHE}	-	13	ACS Appl. Mater. Interfaces 2018, 10, 26178
$Ni_{50}Fe_{50}$@N-CNTs	Ni:Fe=1:1	0.93 V_{RHE}	-	14	ChemCatChem 2019, 11, 5994

Table S6. The ORR performances of $NiFe_{n-1}$@FeNC and pyridinic FeN_4C.

Catalyst	ORR onset potential	pH	Reference
$NiFe_{n-1}$@FeNC	1.05 V_{RHE}	13	This work
pyridinic FeN_4C	0.32 V_{RHE}	13	J. Am. Chem. Soc. 2022, 144, 18144–18152

Table S7. Fitted E (eV)-U (V vs. SHE) quadratic relationships of $Ni_{n-1}Fe@NiNC$.

	Fitted E-U quadratic relationships
Slab	$E = -1.408U^2 - 2.375U - 522.008$
*OH	$E = -1.444U^2 - 2.049U - 531.185$
*O	$E = -1.672U^2 - 1.734U - 525.440$
*OOH	$E = -1.459U^2 - 2.225U - 535.990$

Table S8. The calculated charges (q) and work functions (Φ) of the initial states (is), transition states (ts), and final states (fs) during stage 1 and stage 2 of AIMD simulation. The charges are obtained by calculating the Bader charges of the corresponding structures. All parameters are needed in the constant corrections and charge-extrapolations.

	q_{is} (e)	q_{ts} (e)	q_{fs} (e)	Φ_{is} (eV)	Φ_{ts} (eV)	Φ_{fs} (eV)
Stage 1	0.26	0.72	0.77	4.43	4.18	4.37
Stage 2	0.77	0.69	0.84	4.37	4.49	4.86

Table S9. The free energy changes and barriers of stage 1 and stage 2 of AIMD simulation, ΔE means the values are obtained from the constant charge calculations (AIMD simulation), $\Delta E'$ means the values are obtained from the constant corrections. According to the constant potential correction method, $\Delta E' = \Delta E + 0.5*\Delta q*\Delta\Phi$, $\Delta E_{corr} = 0.5*\Delta q*\Delta\Phi$. All E and Φ are listed in eV, q in the atomic unit t, |e|.

	Δq(is→ts)	$\Delta\Phi$(is→ts)	Δq(is→fs)	$\Delta\Phi$(is→fs)	ΔE_{corr}(is→ts)	ΔE_{corr}(is→fs)	ΔE(is→ts)	ΔE(is→fs)	$\Delta E'$(is→ts)	$\Delta E'$(is→fs)
Stage 1	0.46	-0.25	0.51	-0.06	-0.06	-0.02	0.24	0.13	0.18	0.11
Stage 2	-0.08	0.12	0.07	0.49	0.00	0.02	0.19	0.16	0.19	0.18

Table S10. The free energy changes and barriers of stage 1 and stage 2 of AIMD simulation under 0 V_{RHE}, 0.8 V_{RHE}, and 1.5 V_{RHE} (pH=14). According to the charge-extrapolation method, $\Delta E'' = \Delta E' - \Delta q(\Phi - \Phi_{is})$. $\Phi = 4.57$ under 0.8 V_{RHE} at pH=14, the absolute potential of SHE is -4.6 V. All E and Φ are listed in eV.

	ΔE_0"(is→ts)	ΔE_0"(is→fs)	$\Delta E_{0.8}$"(is→ts)	$\Delta E_{0.8}$"(is→fs)	$\Delta E_{1.5}$"(is→ts)	$\Delta E_{1.5}$"(is→fs)
Stage 1	0.48	0.45	0.12	0.04	-0.21	-0.32
Stage 2	0.14	0.22	0.20	0.17	0.26	0.12

References

1 G. Kresse and J. Furthmüller, *Comput. Mater. Sci.*, 1996, **6**, 15-50.
2 J. P. Perdew, K. Burke and M. Ernzerhof, *Phys. Rev. Lett.*, 1996, **77**, 3865-3868.
3 S. Grimme, J. Antony, S. Ehrlich and H. Krieg, *J. Chem. Phys.*, 2010, **132**, 154104.
4 G. Henkelman, B. P. Uberuaga and H. Jónsson, *J. Chem. Phys.*, 2000, **113**, 9901-9904.
5 P. Xiao, D. Sheppard, J. Rogal and G. Henkelman, *J. Chem. Phys.*, 2014, **140**, 174104.
6 K. Mathew, V. S. C. Kolluru, S. Mula, S. N. Steinmann and R. G. Hennig, *J. Chem. Phys.*, 2019, **151**, 234101.
7 K. Mathew, R. Sundararaman, K. Letchworth-Weaver, T. A. Arias and R. G. Hennig, *J. Chem. Phys.*, 2014, **140**, 084106.
8 Z. Duan and G. Henkelman, *ACS Catal.*, 2019, **9**, 5567-5573.
9 V. Wang, N. Xu, J.-C. Liu, G. Tang and W.-T. Geng, *Comput. Phys. Commun.*, 2021, **267**, 108033.
10 S. Nosé, *J. Chem. Phys.*, 1984, **81**, 511-519.
11 W. G. Hoover, *Phys. Rev. A*, 1985, **31**, 1695-1697.
12 K. Chan and J. K. Nørskov, *J. Phys. Chem. Lett.*, 2015, **6**, 2663-2668.
13 K. Chan and J. K. Nørskov, *J. Phys. Chem. Lett.*, 2016, **7**, 1686-1690.
